# Single-cell genomics reveals population structures from *in vitro* evolutionary studies of *Salmonella*

**DOI:** 10.1099/mgen.0.000871

**Published:** 2022-09-20

**Authors:** Matt Bawn, Johana Hernandez, Eleftheria Trampari, Gaetan Thilliez, Christopher Quince, Mark A. Webber, Robert A. Kingsley, Neil Hall, Iain C. Macaulay

**Affiliations:** ^1^​ Earlham Institute, Norwich Research Park, Norwich, NR1 7UZ, UK; ^2^​ Quadram Institute, Norwich Research Park, Norwich, NR4 7UQ, UK; ^3^​ Medical School, University of East Anglia, Norwich Research Park, Norwich, NR4 7UA, UK; ^4^​ School of Biological Sciences, University of East Anglia, Norwich, Norfolk, UK

**Keywords:** evolution, genomics, salmonella, single-cell

## Abstract

Single-cell DNA sequencing has the potential to reveal detailed hierarchical structures in evolving populations of cells. Single cell approaches are increasingly used to study clonal evolution in human ageing and cancer but have not yet been deployed to study evolving clonal microbial populations. Here, we present an approach for single bacterial genomic analysis for *in vitro* evolution experiments using FACS isolation of individual bacteria followed by whole-genome amplification and sequencing. We apply this to the experimental evolution of a hypermutator strain of *Salmonella* in response to antibiotic stress (ciprofloxacin). By analysing sequence polymorphisms in individual cells from populations we identified the presence and prevalence of sub-populations which have acquired polymorphisms in genes previously demonstrated to be associated with ciprofloxacin susceptibility. We were also able to identify that the population exposed to antibiotic stress was able to develop resistance whilst maintaining diversity. This population structure could not be resolved from bulk sequence data, and our results show how high-throughput single-cell sequencing can enhance experimental studies of bacterial evolution.

## Data Summary

The authors confirm all supporting data and methodologies have been provided within the article or through supplementary data files. Raw sequencing data for this work is available from NCBI under the accession PRJEB40189.

**Impact Statement**

Recent advances in genomic technologies have highlighted a previously unobserved level of microbial diversity. Measuring this diversity at single-cell resolution, both within and between populations, is a potentially powerful tool to explore the mechanisms of bacterial disease and antimicrobial resistance. In this study we applied single-cell whole genome sequencing and *in vitro* evolution of populations of *Salmonella* under weak antibiotic stress and show that even under weak selection bacteria were able to evolve antimicrobial resistance. Furthermore, these populations exhibited increased population structure compared to antibiotic free cultures. This suggests small antibiotic concentrations could not only promote the emergence of resistance but also increase population diversity, which would have implications for adaptation to other stressors and environments. Our work demonstrates the power of single-cell genomics for the study of bacterial populations.

## Introduction

A long-term evolution experiment in *E. coli* has emphasised the insights that can be gained from the study of evolutionary processes in bacteria *in vitro* [1]. Experimental evolution studies have the power to transform our understanding of the molecular mechanisms underpinning the emergence of phenotypic traits including resistance to antimicrobial compounds [2]. These experiments are typically analysed by either characterising individual isolates, or by assessing the genetic changes occurring in populations through the bulk sequencing of cultured samples. Neither approach can fully resolve the genetic heterogeneity present within bacterial communities; to do so requires single-cell genomic analysis with sufficient coverage and resolution to capture single-nucleotide polymorphisms (SNPs).

Recent developments in genomic technologies have revealed a previously unknown diversity of microbial life and led to the classification of new bacterial and archaeal phyla [3, 4]. Parallel to this, new imaging techniques have highlighted previously hidden heterogeneity and dynamics in the response of individual cells within a population to environmental stress such as antibiotics [5, 6]. Single-cell whole genome sequencing can increase our understanding of these complex systems and responses by isolating the contribution of individual genomes [7, 8].

Many bacterial species cannot be grown outside of their natural environments, and though metagenomic analysis can reveal the genomic composition of whole populations or communities, understanding evolutionary dynamics at the scale of sub-populations or strains remains challenging. Single-cell whole genome sequencing is critical to addressing this challenge as recently demonstrated in the intestinal microbiome [9] and in environmental populations [10, 11].

While selection of individual colonies and subsequent sequencing provides a measure of within-population diversity, this is restricted to culturable species, and even then, selection may occur during clonal expansion, thus providing a skewed representation of the starting population. As each genome is sequenced individually, single-cell whole genome sequencing can also enable haplotype phasing in a manner that is not confounded by complex population structures and dynamics.

**Fig. 1.**
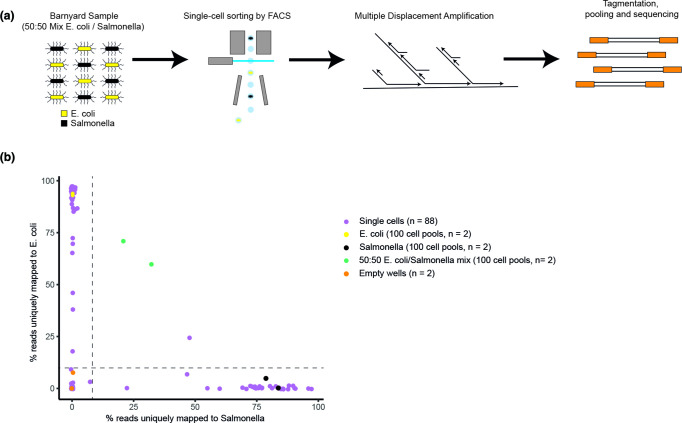
a) Overview of methodology. Individual bacteria, in this example from ‘barnyard’ sample containing a 50 : 50 mixture of *E. coli* (yellow) and *Salmonella* cells (black), were sorted from liquid culture samples by FACS, followed by MDA and Illumina sequencing. b) Barnyard analysis in which the percentage of reads mapping to each genome for each single cell (purple), empty wells (orange) and multi-cell controls (black - *Salmonella *, Yellow - *E. coli* and green - mixed), are shown.

Single-cell studies on bacterial systems highlighted the applicability of Multiple Displacement Amplification (MDA [12]) to provide enough material for genome analysis [13] and the potential to sequence uncultured isolated cells [14]. Subsequent studies focused on *de novo* genome assembly from single cell sequencing [15, 16] and technology development to facilitate this [17]. Nevertheless, the majority of single-cell genomic technology development to date has involved eukaryotic systems, and high-throughput effective solutions dedicated to microbial systems remain a largely unmet need [18]. Whilst some studies have been able to identify genomes from single bacterial cells to identify the presence of mixed populations or specific species in environmental niches [10, 19, 20], the application of single-cell sequencing to resolve population structure in an evolving community has yet to be explored. Here, we present an approach to investigate the structure of bacterial populations after selection for antimicrobial resistance and provide new information about the dynamics within populations exposed to the antibiotic ciprofloxacin. We used Fluorescence Activated Cell Sorting (FACS) followed by MDA to isolate and sequence genomes from hundreds of individual bacterial cells from populations after growth in control or antibiotic stress conditions (Fig. 1a).

## Methods

### Single bacterial isolation by FACS

Liquid cultures of bacterial cells were grown in Luria Bertani broth (LB) at 37 °C and diluted in PBS. Bacterial cells were sorted by size (FSC-A) and granularity (SSC-A) using Fluorescence Activated Cell Sorting (FACS, BD FACS Melody). Individual cells were sorted into wells of a 96-well plate, and multi-cell (100 cell) control wells were also sorted.

**Barnyard experiment** a 50 : 50 mix of *Salmonella* ((* Salmonella enterica* subsp. enterica serovar Enteritidis str. P125109 [21]) and *E. coli* (MG1655) [22]) cultures was prepared and individual cells meeting within a bacterial gate (FSC/SSC) were sorted from this mixture – although the MG1655 cells used here constitutively express YFP this was not used as marker for sorting. Additional 100 cell controls were sorted from this mixed culture, as well as from unmixed *Salmonella* and *E. coli* cultures. In total 88 cells were sorted from the 50 : 50 mixture, as well as two unmixed E. coli samples (100 cells), two unmixed *Salmonella* samples (100 cells) and two bulk samples of the 50 : 50 mixture (100 cells). Two empty wells were also processed as negative controls.

### Whole genome amplification (WGA)

WGA of single cells was performed by multiple displacement amplification using the REPLI-g Single Cell kit (Qiagen, Valencia, CA) as per the manufacturer’s instructions but at one quarter of the recommended volumes. The amplified product was cleaned up with AMPure XP beads on a robotic liquid handling platform (Biomek Nx, Beckman Coulter), quantified by Qubit (Thermo Scientific) and diluted to approximately 1 ng µl^−1^ before proceeding with library preparation.

### Single-cell sequencing library preparation

Sequencing libraries were prepared with Nextera XT kit (Illumina) as per the manufacturer’s instructions but at one quarter of the recommended volumes. The library product was pooled and again cleaned up using Ampure XP beads. The pooled library size was examined using a Bioanalyzer (Agilent) using the High Sensitivity DNA kit and quantified using the KAPA Library Quantification Kit (Roche).

### Barnyard experiment genome sequencing and data analysis

Paired-end (150 bp) sequencing was performed on the Illumina MiSeq platform using a Nano flow cell. For the barnyard experiment, sequencing reads were mapped to *E. coli* K12- MG1655 (genbank U00096.2 [23]) and *S*. Enteritidis strain P125109 (this work see below) reference genomes using bbsplit in bbmap-38.06 [24]. The percentage of reads uniquely mapping to each reference was plotted (Fig. 1b). A conserved site reference was made by aligning the *S*. Enteritidis and *E. coli* genomes using nucmer-3.1 with maximum gap of 500 and minimum gap 100 nucleotides. Regions of greater than 75% nucleotide identity were taken from the *E. coli* fasta sequence and concatenated with 200 N spacers into a single sequence. The *breseq* pipeline (below) was then used for variant calling. We identified 701 variant sites that were used to construct a fasta sequence alignment. The nucleotide sequence was converted to a trinary numeric matrix using 0 for non-coverage positions, 1 for coverage and 100 for a variant position. The Euclidean distance matrix of the trinary input of the 100 sorted cell samples (*E. coli S*. Enteritidis and mixed) was analysed by PCA using the factoextra package in R (Fig. S5e).

### Bacterial cell culture and antibiotic stress

Two *S*. Enteritidis strains were used in this study, P125109 [AM933172.1] [21] and its isogenic *mutS* deletion mutant (included to potentially see more variation in a short period due to an elevated mutation rate). The MIC of ciprofloxacin was determined for both strains as being 0.08 mg l^−1^. Both strains were repeatedly passaged in the presence and absence of 0.5 × the MIC of ciprofloxacin in LB broth, twice daily (am and pm) over 2 weeks. Parallel lineages were run with two dilution factors (1 : 1000 and 1 : 100 000). The experiments completed between ~150 and ~300 generations (depending on the inocula). After each passage an aliquot (0.5 ml) of each culture was stored with 15 % glycerol and frozen to provide an archive of each time point for all lineages. The finally passaged lineages from the 1 : 1000 dilution series were sampled for the single cell analysis. Single-cell sorting, WGA and library preparation was performed as for the barnyard experiment but the sorted *S*. Enteritidis/*mutS* cells were sequenced using NovaSeq 6000 platform (SP flow cell, PE 150 bp reads).

### AMR phenotyping

Susceptibility testing used the broth and agar dilution methods with Mueller-Hinton media following the guidelines and interpretation criteria provided by EUCAST (http://www.eucast.org/ast_of_bacteria/).

### Bulk DNA extraction

Cells were grown overnight at 37 °C at 200 r.p.m. in LB broth. The overnight culture was pelleted by centrifugation at 3 220 g for 15 min. The supernatant was discarded and the pellet was resuspended in 9 ml of sterile PBS. Cells were lysed by adding 1 ml of 20% SDS and 50 µl of a 20 mg ml^−1^ proteinase K solution before incubation at 37 °C for 1 h.

For DNA purification, 1 vol of phenol:chloroform:isoamyl alcohol mixture (25 : 24 : 1) was added to 1 vol of cell lysate and centrifuged at 3 220 ***g*** for 5 min. The aqueous phase was recovered with a cut-end 1 ml tip and transferred to a new clean tube. A volume of chloroform:isoamyl alcohol mixture (24 : 1) was added to the aqueous phase, and the tube was mixed gently by inverting until the content turned milky. The tube was centrifuged at 3 220 ***g*** for 5 min and the aqueous (top) phase was recovered once again with a 1 ml cut-end tip and transferred to a new tube. To precipitate the DNA, 1/10 volumes of 3 M sodium acetate pH 5.2 and 2.5 volumes of ice-cold absolute ethanol were added to the tube before mixing gently by inverting until DNA precipitates. A Pasteur pipette hook was used to spool the DNA pellet and transfer it in a 2 ml tube containing 80% ethanol for a 5 min wash. The DNA pellet was then air dried for 5 min on the hook before being resuspended in 200 µl of ultrapure water, by incubation at room temperature overnight.

### Heterogeneity of susceptibility

To examine the heterogeneity of susceptibility within a population, 96 individual colonies were isolated from *mutS* lineages (exposed and control) and the MICs of ciprofloxacin and nalidixic acid were determined to identify the prevalence of resistance within the population. This confirmed selection of resistance in the exposed lineage, but also identified heterogeneity within the population with 13.6% of individual isolates being resistant (MIC >0.06 mg l^−1^) to ciprofloxacin in the exposed lineage (Fig. S1), compared to none in the control. This data confirmed there was heterogeneity in the population and therefore the samples were a suitable test set for analysis by single cell sequencing.

### Bulk Illumina sequencing

Ten reference samples were sequenced on the Illumina NovaSeq platform with a PCR-free library preparation and generating 150 base paired end reads. Genomes were sequenced to an average depth of around 2700×.

### Reference genome

The chromosomal sequence of *Salmonella enterica* subsp. enterica serovar Enteritidis str. P125109 (AM933172) [21] annotated using Prokka (version 1.11) [25] was used as a reference genome.

### Variant calling from bulk data

Read mapping to the reference and variant calling was performed using the breseq pipeline [26], developed for the long-term *E. coli* evolution experiment. This pipeline uses bowtie2 [27] for mapping and reports the fraction of reads that align to the reference genome. Polymorphisms present in at least one percent of reads with a read coverage of 10 on each strand at the variant position were called. Variants only seen on one strand were removed. The *breseq* pipeline also calculated various mapping and coverage statistics.

### Single-cell variant calling

Read mapping to the reference and variant calling was performed using the breseq pipeline, as above. Polymorphisms present in at least one percent of reads with a read coverage of 10 on each strand at the variant position were called. Variants only seen on one strand were removed.

### Fixed snp sites in coding regions

Single cell genomes exhibiting variation (above) were considered. The non-unique fixed SNP sites (SNPs present at a frequency greater than 0.9 and seen in more than one single cell genome) in the single-cell genomes were determined leading to 3452 SNPs at 357 unique locations and ultimately 315 SNP locations within genes. The alignment coverage at each of these positions was determined from the *breseq* bam files using breseq. Each SNP location could then be classified as SNP, reference or low-coverage for each single cell genome. Genomes with more than the mean plus one standard deviation positions out of the 315 candidate sites lacking sufficient coverage for SNP calling were excluded for phylogenetic reconstruction and population structure leading to 177 single cell genomes from all populations remaining for phylogenetic reconstruction.

### All SNP sites

SNP sites including intergenic SNPs present at a frequency >0.9 were selected. G-A or C-T transitions seen in more than one single cell were retained resulting in 1807 variant sites. The alignment coverage at each of these positions was determined from the *breseq* bam files using Samtools-1.9. Low coverage genomes were removed from the alignment as above leading to 216 single cell genomes from all populations remaining.

### Phylogenetic reconstruction from single-cell data

A sequence alignment of variant sites from above was used to generate a maximum likelihood phylogenetic tree, for each culture, with RAxML using the GTRCAT model implemented with an extended majority-rule consensus tree criterion [28]. RHierBaps (an R implementation of hierarchical Bayesian analysis of Population Structure) [29] was used to estimate population structure from the sequence alignment using two nested levels of molecular variation and up to 20 populations.

### Principal component analysis (PCA)

PCA was performed using R package factoextra. The sequence alignment used for phylogeny reconstruction for each case was converted to a three-level numeric matrix with values of 100, 1 and 0 for SNP, non-variant, and missing-data respectively, and then converted to a Euclidean distance matrix for which the principal components were determined. The fviz_pca_ind function was used to generate a PCA plot.

### Rarefaction curves

To estimate the number of SNPs that would have been observed at smaller single cell sample numbers, the following subsampling strategy was used: synthetic collections of cells were generated by sampling at random without replacement from the observed single cell SNP profiles. The number of unique SNPs in these subsampled collections were then counted. This process was repeated 1000 times for every collection size between one and the actual single cell sample number. The median and 95% confidence intervals in the subsampled SNP numbers were then calculated. To estimate the total SNP diversity we fitted Michaelis-Menten saturating curves to the median estimates using non-linear least squares as implemented in the R nls function. The total SNP diversity as the Michaelis-Menten curve prediction at infinite sample size was estimated. This procedure was repeated for all four treatments using the filtered SNPs although we failed to fit a Michaelis-Menten curve for the WT strain in the presence of ciprofloxacin.

## Results

To demonstrate the ability to sort individual bacteria, we performed a ‘barnyard’ experiment, sorting single events from an artificially inoculated 1 : 1 mixture of *E. coli* and *S. enterica* serotype Enteritidis PT4 isolate P125109 (*S*. Enteritidis) into a 96 well plate, followed by whole genome amplification and sequencing. Individual cells predominantly had reads mapping to either one or the other genome, indicating that individual bacteria can be sorted from a mixture with a low rate of doublet cells (Fig. 1b). Empty wells generated negligible amounts of mapping reads, while deliberately mixed mini-bulk wells showed a mixture of reads mapping to each genome.

To demonstrate the ability of this approach to detect genomic heterogeneity and study population structure, we generated test populations founded by a wild-type (WT) *Salmonella enterica* subsp. enterica serovar Enteritidis (herein referred to as *S.* Enteritidis), or its isogenic hypermutator strain with an internal and in-frame *mutS* deletion mutant (*mutS*). This specific *mutS* mutation was previously identified in a strain that caused a chronic infection in an immunocompromised patient spanning more than a decade and that evaded antimicrobial therapy leading to selection of antimicrobial resistant sub-populations [21]. Both strains were repeatedly passaged in the presence or absence of half the Minimum Inhibitory Concentration (MIC) of ciprofloxacin twice a day over 2 weeks to give ~150 generations. We chose this experimental model as multiple genes are known to impact ciprofloxacin susceptibility at the SNP level and ciprofloxacin resistance mechanisms are well characterised although the succession of selection of different mutations remains unclear [30]. We sampled populations of both the WT and *mutS* strains at the end of the exposure series. We expected to see a difference in diversity between drug-exposed and naive populations in these conditions with selection in the former potentially imposing a bottleneck on drug-sensitive lineages.

Measurement of the MICs of ciprofloxacin against the final populations revealed no change in ciprofloxacin susceptibility in untreated lineages but emergence of resistant mutants in all populations exposed to the antibiotic (Table S1). Isolation and susceptibility testing of randomly selected individual colonies from the final populations confirmed the emergence of resistant variants in the exposed lineages. This analysis also allowed us to determine the heterogeneity of ciprofloxacin susceptibility within the population with 13.6% of isolates being resistant (MIC >0.06 mg l^−1^) to ciprofloxacin in the exposed lineage (Fig. S1).

**Fig. 2.**
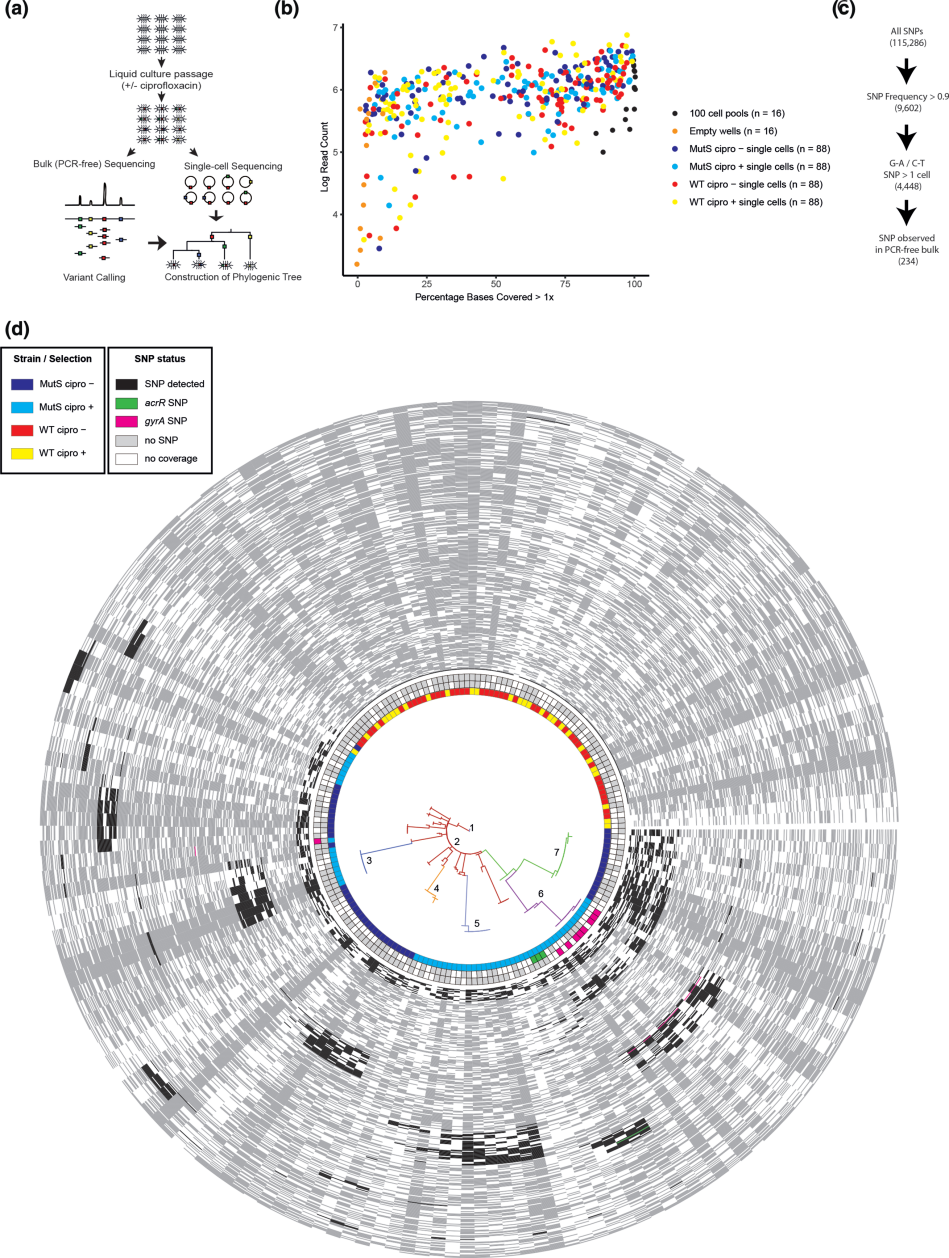
a) Experimental overview, *S*. Enteritidis was cultured in the absence and presence of sub-inhibitory concentrations of ciprofloxacin for around 150 generations. Bulk, PCR-free sequencing and single-cell sequencing of individual cells from the liquid culture were then performed. **b**) Coverage plot for all whole genome amplified samples. **c**) Overview of SNP filtering method for variants called from sorted cells. d) Phylogeny and population structure of all single cell genomes evolved in the absence and presence of ciprofloxacin using 315 high confidence variant sites. The colour of tree branches indicates the determined population group. Three rings outside the phylogeny highlight; population group and the presence of SNPs in *acrR* and *gyrA* respectively. The heatmap outside the phylogeny and inner rings indicates the coverage and SNP status of each site in each sample used to construct the phylogeny (red: SNP, grey: coverage but no variant, white: no coverage). In the figure on the left, SNPs also present in the absence of ciprofloxacin are indicated in blue. The SNPs in *gyrA* and *acrR* are also highlighted.

We sorted and sequenced 352 single WT and *S*. Enteritidis *mutS* cells, from populations cultured in the presence or absence of ciprofloxacin (88 cells from each group), as well as mini-bulk (100 cells) and empty wells for each experimental group. In parallel, we performed high-coverage Polymerase Chain Reaction (PCR) free sequencing of the cultured populations to determine high-confidence variant calls (Fig. 2a).

In many of the single-cell samples, over 40% of bases of the *S*. Enteritidis genome were sequenced, and in some cases, near-complete genome coverage was obtained from single cells (Fig. 2b). The mean percentage of reads that aligned to the *S*. Enteritidis reference genome for single-cells was 69 %, with a mean coverage of 64×. PCR-free bulk libraries were sequenced to a depth of >1 000×. In the bulk sequencing data 2753 SNPs were called at 1500 unique genomic sites for the four samples, with more transitions seen in the *mutS* (mean proportion=0.53) than in the WT strain (mean proportion=0.23) (Fig. S2a). This pattern is expected due to the *mutS* deletion in the hypermutator and was previously seen in the *mutS* hypermutator strain of *S*. Enteritidis that evolved within an immunocompromised patient [21].

In the corresponding single cell genome data, 115 286 raw SNPs were called at 99 617 unique genome sites. SNPs were divided into 12 categories (Fig. S2) to assess possible error profiles due to single cell genome amplification and sequencing. In this work we decided to concentrate on the use of SNPs only to infer sub-populations. While insertions and deletions could also have been used, they comprised less than ten percent of total variants and their inclusion would have necessitated a revised variant filtering approach. SNP profiles were similar for all four samples and distinct to those seen in bulk sequencing. Single-cell SNPs were dominated by G-A and C-T transitions (mean proportion 0.42) and C-A and G-T transversions (mean proportion 0.38) (Fig. S2b). The prevalence of these transitions and transversions in multiple displacement amplification (MDA) has been previously described and attributed to deamination (transitions) or alkylation (transversions) during cell lysis [31, 32].

We filtered the single-cell SNP data to remove as many false positives as possible. As true single-cell SNPs should be monoallelic, single cell SNPs present at frequencies below 0.9 were then removed (Fig. S3), comprising 105 684 SNPs (92% of initial single cell SNPs) at 93 239 positions, leaving 9602 SNPs at 6687 positions remaining. Inspection of the remaining SNP profiles showed that there was still an increased signal from G-A and C-T transitions when compared to the profile from the bulk sequencing - 4981 more G-A and C-T SNPs than A-G and T-C SNPs in the single-cell samples. Assuming the true number of transition types should be approximately the same as seen in the bulk data, we inferred that many of these additional SNPs were false positives. To remove potential false positives from further analysis, G-A and C-T SNPs seen in only one single-cell genome were removed, leaving 4448 SNPs at 1536 positions. The vast majority of SNPs present in our samples are expected to be rare, having arisen in the last generation of the experiment [33] and the site frequency spectra of SNPs confirms this (Fig. S4a–d). Of the filtered sorted-cell SNP positions, 234 (16 %) also contained SNPs in the bulk sequence data. The mean frequency of bulk SNPs with positions also seen in the single cell SNPs is 0.18, compared with 0.03 for SNPs at positions not seen in single cells, reflecting the skew towards observation of rare SNPs in the data. A maximal intersection of bulk with sorted SNPs of 71% was determined by increasing the threshold of observation to a frequency of 0.4 for both bulk and sorted cell data (Fig. S4e). We used subsampling without replacement to estimate the number of filtered SNPs with confidence intervals we would have seen at smaller single cell sampling numbers (Fig. S5). This allows us to both compare SNP diversities whilst correcting for sampling numbers and estimate the uncertainty in those predictions. This shows that our sampling was sufficient to demonstrate a significantly higher SNP number for the hypermutator strain in the presence of ciprofloxacin although we could not say the same for the impact of the antibiotic on the WT strain. The fact that the hypermutator sampling curves are flattening suggests that we have accessed a significant fraction of the SNP diversity (estimated 54 and 59% of SNPs without and with ciprofloxacin respectively) again the same is not true for the WT strains where although fewer SNPs were observed we see no evidence that the number of SNPs is saturating (Fig. S5). This could be due to the WT population consisting of a small number of dominant but similar lineages and multiple low frequency haplotypes, and the hypermutator being dominated by a large number of relatively high frequency genetically distinct clones. This hypermutator scenario is consistent with the observed tree structure.

To investigate the utility of the increased resolution that this approach gives to population analyses we used a set of 315 high confidence SNPs in coding sequences of the single-cell genomes to elucidate phylogeny and population structure in the WT and *mutS* strain in the absence and presence of ciprofloxacin (Fig. 2d).

Since one of the experimental conditions was the presence of ciprofloxacin, we expected to observe SNPs in genes previously demonstrated to be associated with ciprofloxacin resistance. Examining the variants seen in the bulk sequencing data in the hypermutator strain (recovered from the population passaged with a 1 : 1000 dilution rate), SNPs were present in *gyrA* D87G (frequency 0.14), *acrR* T12A (frequency 0.06), *envC* G347S (frequency 0.014) and *ompX* A9V (frequency 0.011). In the corresponding single-cell data the *gyrA* D87G mutation was seen in 11 out of 88 (a frequency of 0.125) *mutS* cells grown in the presence of ciprofloxacin (Fig. 2d). These frequencies are in agreement with those seen in the bulk sequencing data as well as the frequency of resistant isolates experimentally observed in the populations and show that the emergence and prevalence of resistant mutants was accurately detected by the single-cell approach (Fig. S1). The *acrR* T12A SNP was seen in three single-cell genomes (a frequency of 0.034) (Fig. 2d). The *envC* and *ompX* mutations seen in the pooled data at minor frequencies (~0.01) were not observed in the single-cell genomes, likely due to their low frequency.

The population structure analysis of the 315 high confidence variant sites within all four experimental conditions determined seven first-level clades (Fig. 2d). Of these, practically all WT cells were placed into population 7, the further six levels containing cells of the hypermutator strain: population one was the basal population containing cells grown with and without ciprofloxacin. Populations 2 and 4 were predominantly ciprofloxacin negative, and populations 3, 5 and 6 ciprofloxacin positive. Furthermore our analysis is able to place populations in an evolutionary context - for example populations 2 and 3 share several SNPs but population three diverged and contains more total SNPs including the *gryA* mutation. In the context of this experiment this suggests that the common SNPs could be due to adaptation to culturing and indeed a closer examination of the shared SNPs highlights variation in genes associated with cell maintenance and metabolism (Table S2). The phylogenetic and population level analysis was also performed using 1807 putative variant sites in coding and non-coding regions (Fig. S6). More uniquely ciprofloxacin positive than negative populations were again identified, consistent with an unexpected increase in diversity under selection. Ciprofloxacin has been shown to have a mild mutagenic effect which may impact the mutant accumulation seen here although ciprofloxacin induced mutations have been demonstrated to largely be short insertions and deletions rather than SNPs [34]. This greater diversity in the drug-exposed culture suggested that a population exposed to a sub-inhibitory concentration of antibiotic can both develop resistance whilst maintaining overall diversity, since under these conditions no significant bottleneck was imposed on the population. This highlighted the power of single-cell approaches to dissect complexity in cell populations that would not be visible if only bulk sequencing had been performed, in part due to the inability to co-locate SNPs within individual genomes. Several SNPs were present in populations following culture with or without supplementation with ciprofloxacin that are likely due to adaptation to the media. However, some were unique to the *mutS* strain grown with antibiotic, and sub-clones of *gyrA* D87G and *acrR* T12A mutant cells were present as distinct clades on the phylogenetic tree (Fig. 2d). Mutants with very high ciprofloxacin MICs often contain both changes in *gyrA*, and efflux pump expression (as conferred by loss of function of *acrR*), [30] our data demonstrate both changes occurring and being selected in isolation in the populations subjected to ciprofloxacin. The phylogenetic analysis provided an evolutionary roadmap for the bacterial populations and the framework for the imputation of variants in incompletely covered genomes. PCA analyses Fig. S7 using the same input data used to construct phylogenies in Figs 2d and S6 supported the results of our phylogenetic analysis.

## Discussion

Our approach enables the high-throughput and reliable determination of SNPs from individual bacteria within a population, which when combined with *in vitro* evolution experiments can be used to resolve population structure in evolving populations. This method allows greater understanding of population dynamics and will help predict evolutionary trajectories for populations exposed to stresses of interest. A similar *S*. Enteritidis hypermutator strain to that used in these analyses was able to evade antimicrobial therapy over more than a decade of intensive treatment [21]. Our data suggest increased genotypic diversity within a population resulting from antimicrobial therapy failure has the potential to provide a pool of diversity that can lead to a stepwise increase in resistance to future antimicrobial challenge.

Beyond the *de novo* mutation associated with adaptation to sub-inhibitory concentrations of ciprofloxacin we saw several mutations potentially associated with adaptation to the general evolution experiment environment (Table S2). For example, a mutation was found in *frsA*, encoding an esterase. Disruption of this gene has been shown to increase cellular respiration on glucose and several other sugars in *E. coli* and was hypothesised to be part of switch between respiration and fermentation [35]. Mutations were also seen in the putative lipoprotein, *yaiW*. Mutations in this gene have been described in *E. coli* as off target mutations, in response to co-exposure to streptomycin and pesticides [36]. This gene has a similar function to the inner membrane transporter *sbmA*, mutations in which have been proposed to alter cellular permeability and affect uptake of external molecules [37]. A mutation is also seen in the minor curlin subunit *csgB*, disruption of this region is associated with impaired biofilm formation in *Salmonella* Typhimurium [38]. It is therefore, plausible that disruption of these coding regions could confer an advantage to bacteria under the growth conditions experienced during the evolution experiment [39].

In summary, we have demonstrated that single-cell isolation, whole genome amplification, SNP detection and evolutionary analyses from bacterial genomes can be readily performed on hundreds of cells derived from complex liquid cultures. Although here we have applied the method to unlabelled cells, the use of FACS would enable the separation of individual cells on the basis of differentially expressed reporter genes or molecular probe-based detection. Which when coupled with advances in liquid handling and microfluidic technology could enable the rapid scaling of such approaches to incorporate analysis of thousands of individual bacteria. Furthermore, the development of multi-omic approaches such as G&T-seq [40] to be compatible with bacterial transcriptomics would enable the mapping of transcriptional phenotypes to genomic phylogenies, thus revealing the functional consequences of genetic variation at single-cell resolution.

## Supplementary Data

Supplementary material 1

Supplementary material 2
