# Supplementary material for: Single-cell genomics reveals population structures from in vitro evolutionary studies of Salmonella
Source: Microb Genom. 2022 Sep 20;8(9):mgen000871. doi: 10.1099/mgen.0.000871 (PMC9676037; doi:10.1099/mgen.0.000871)
Supplement: Supplementary material 1 [file mgen-8-871-s001.pdf]

Supplementary Figure 1

A

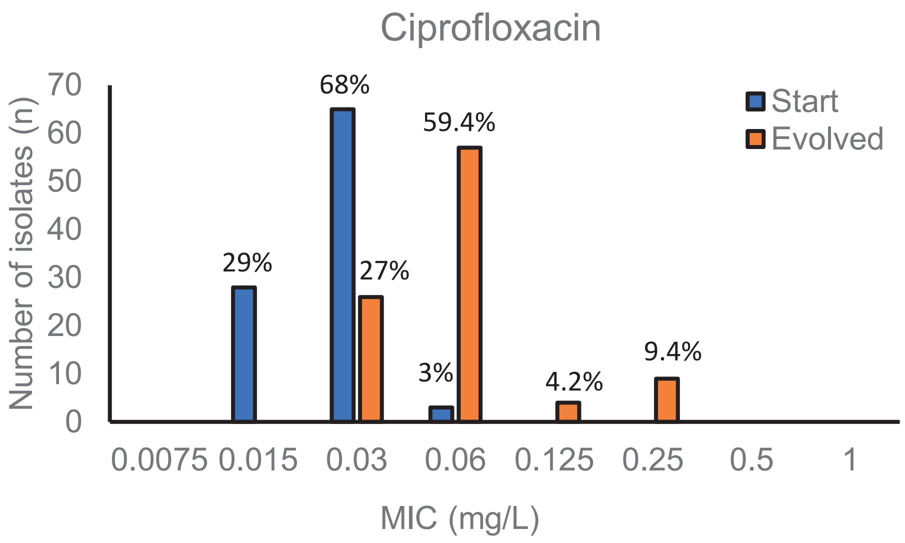


B

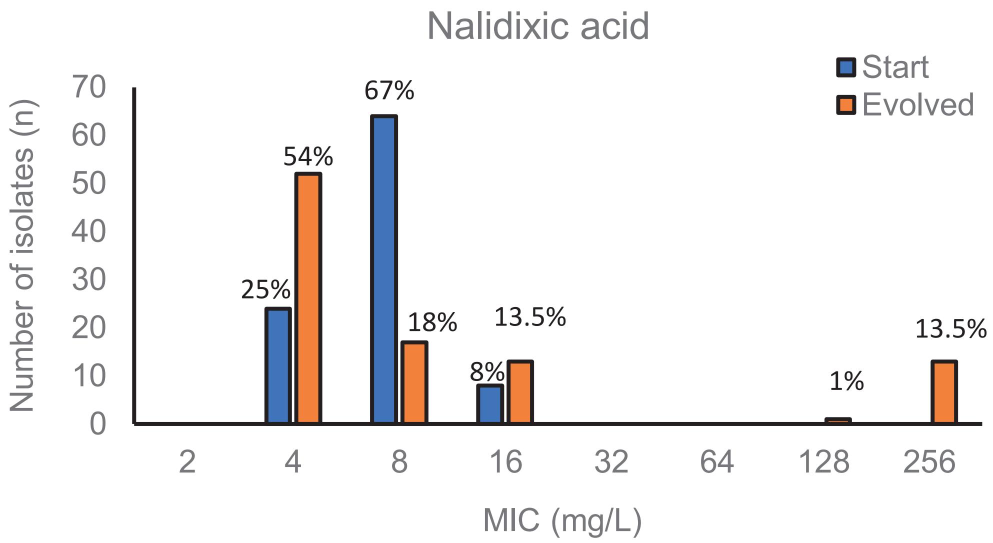


**Supplementary Figure 1** Heterogeneity of susceptibility of isolates to ciprofloxacin within populations. 96 individual colonies were isolated from *mutS* lineages (exposed and control) and the MIC of (A) ciprofloxacin and (B) nalidixic acid against all isolated strains was determined to identify the prevalence of resistance within the population. This confirmed selection of resistance in the exposed lineage, and identified heterogeneity within the population with 13.6% of individual isolates being resistant (MIC > 0.06 mg/L) to ciprofloxacin in the exposed lineage.

Supplementary Figure 2

A

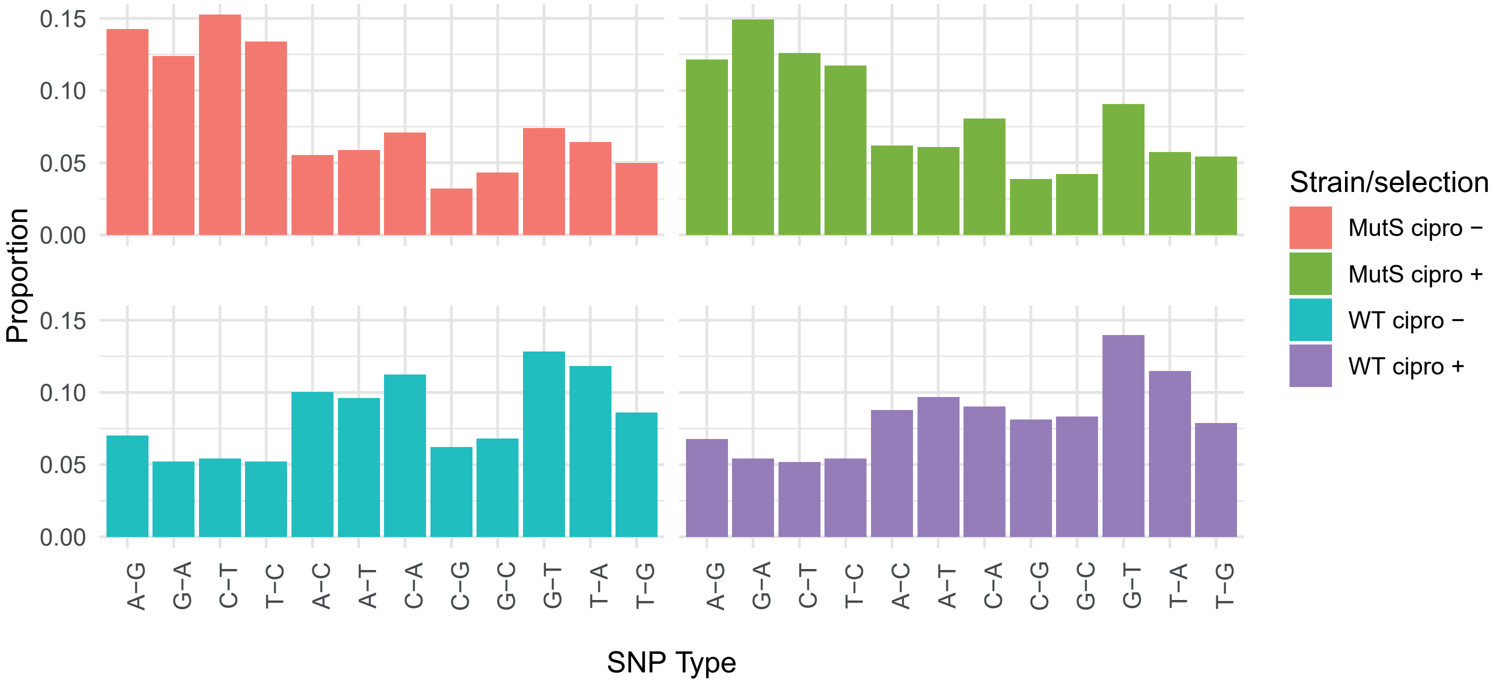


B

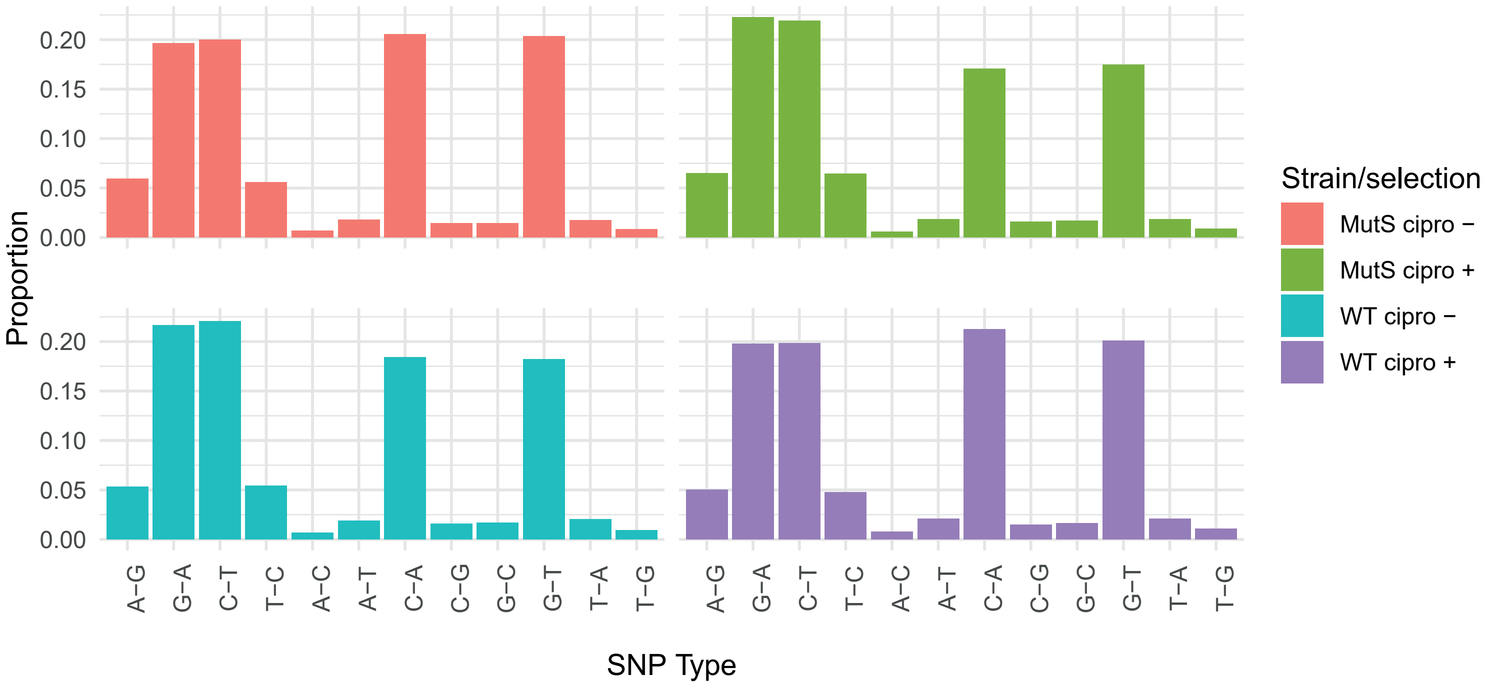


**Supplementary Figure 2** Proportion of SNP types seen in (A) bulk and (B) single cell sequencing before filtering procedure. In the bulk sequencing data 2,753 SNPs are called at 1,500 unique genome sites for the four samples, SNP profiles show differences between the wild-type and hypermutator with more transitions are seen in the hypermutator (mean proportion = 0.53) than in the wild type (mean proportion = 0.23). In the single cell data 115,286 SNPs are called at 99617 unique genome sites. SNP profiles are similar for all four samples and distinct to those seen in bulk sequencing. SNP types are dominated by G-A and C-T transitions (mean proportion 0.42) and C-A and G-T transversions (mean proportion 0.38)

Supplementary Figure 3

A

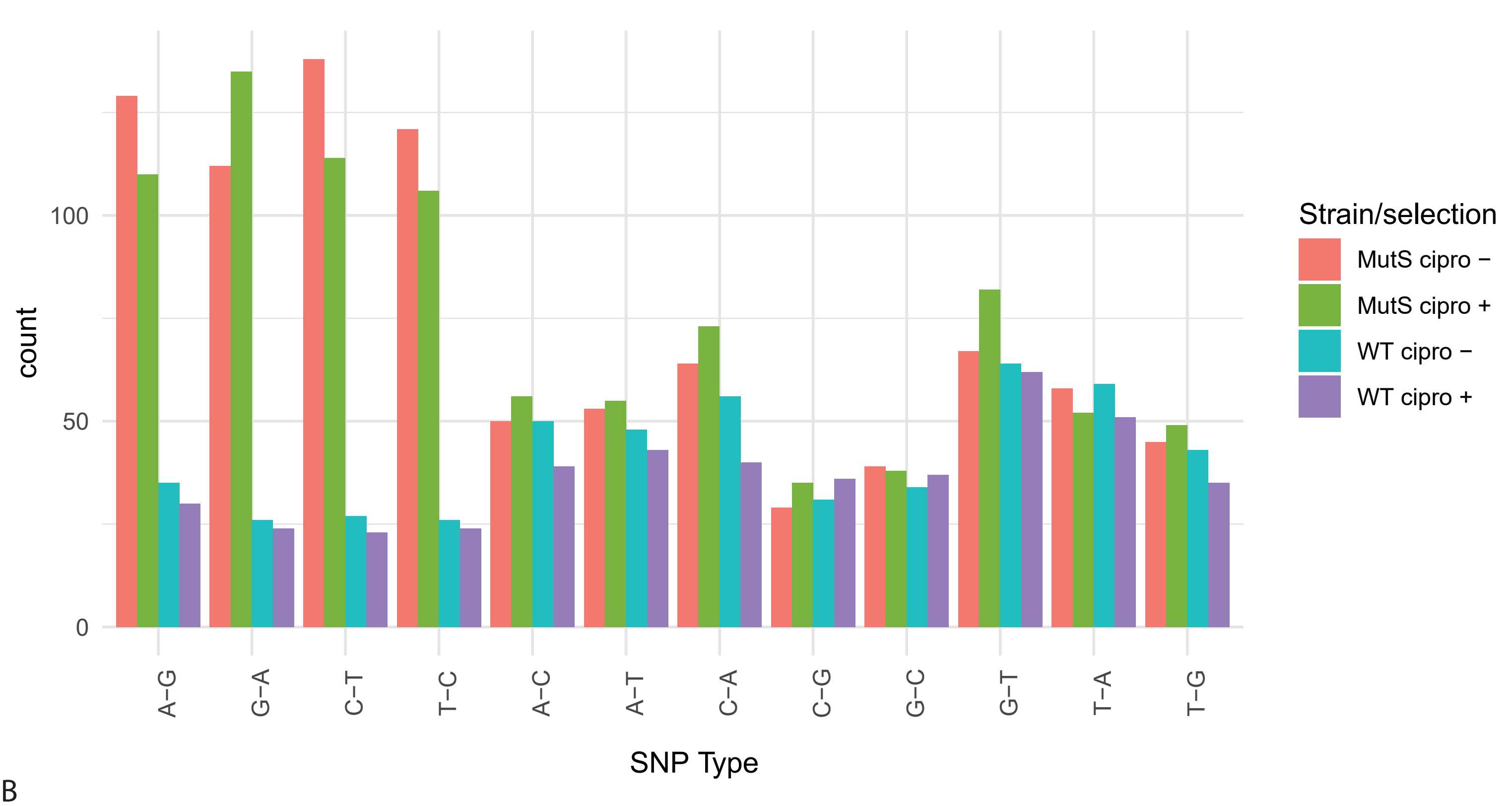


B

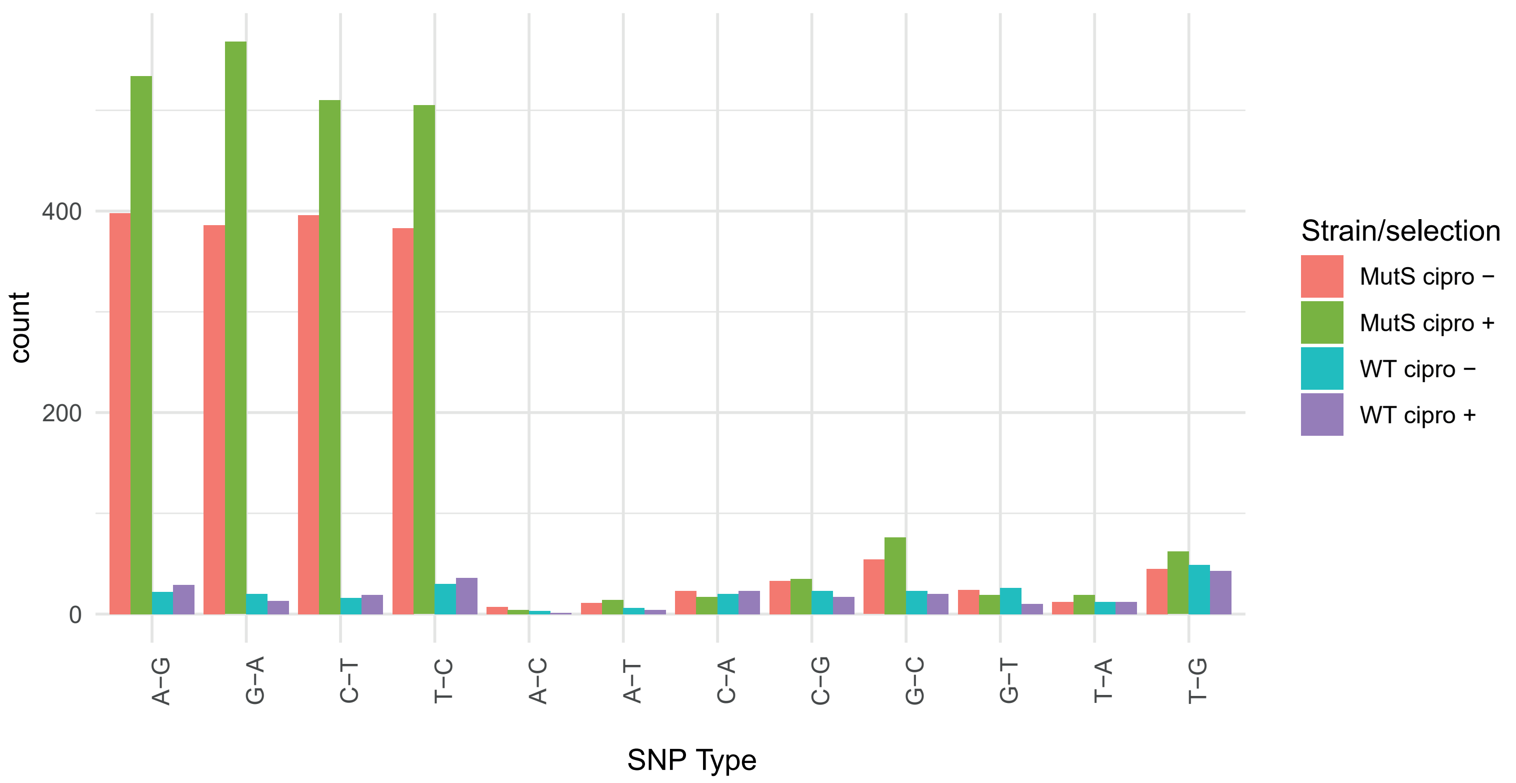


**Supplementary Figure 3** A) SNP profiles of bulk PCR free sequencing and (B) filtered single cell SNPs derived from MDA. Single-cell SNPs were filtered by removing low frequency variants and G-A and C-T SNPs only seen in one single cell genome, resulting in 4,448 SNPs at 1536 positions. Of these SNPs 234 (16%) of locations were also seen in bulk SNPs.

Supplementary Figure 4

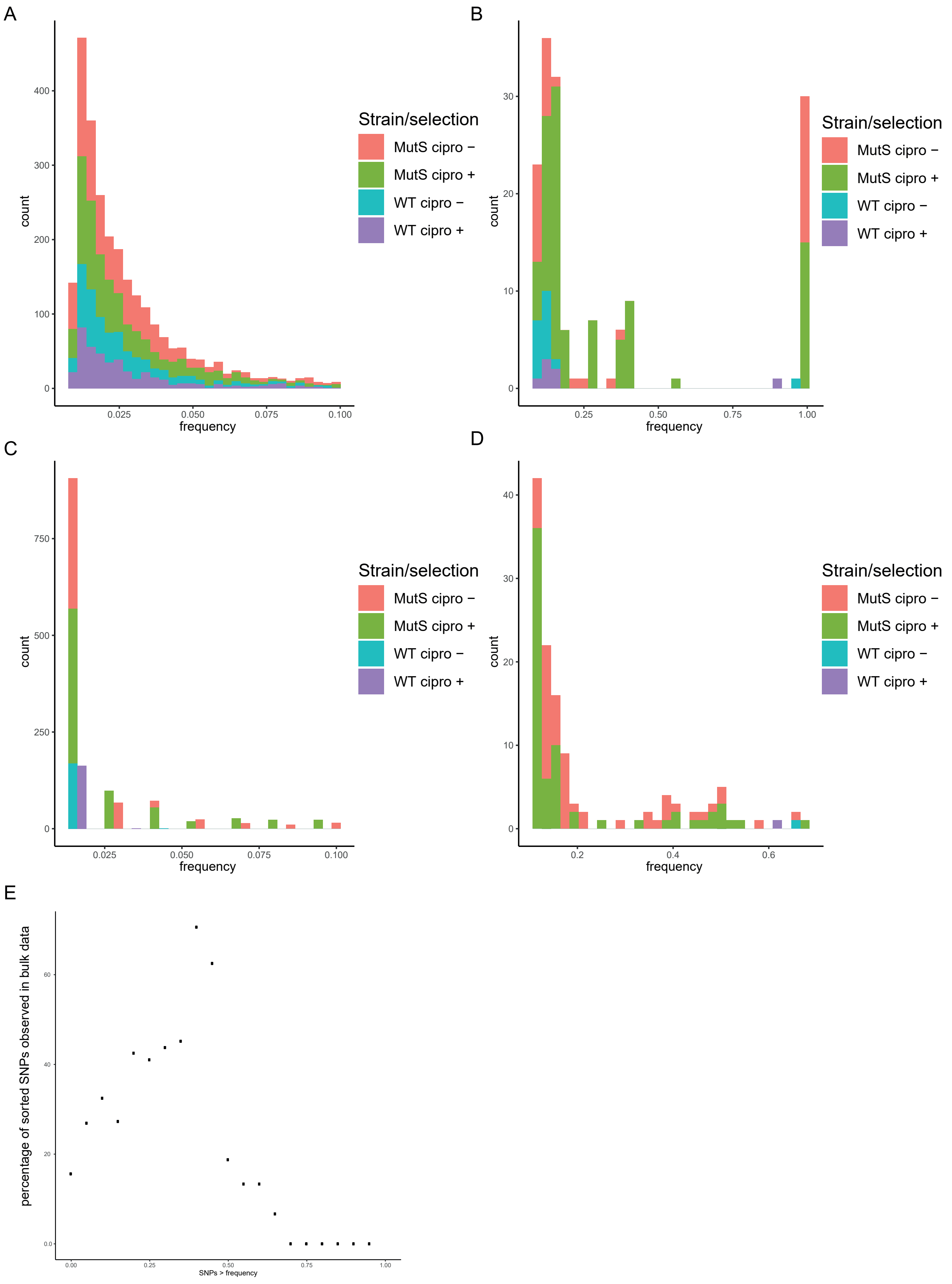


**Supplementary Figure 4** Site frequency spectra of: low (observed frequency of 0-0.1) and high frequency SNPs (observed frequency of 0.1 and above) SNPs seen in bulk and sorted cell data.  A) Bulk low B) Bulk high, C) sorted filtered low D) sorted filtered high E) intersection of SNPs in bulk and sorted cells with variation in observed frequency. For sorted cells the reported frequency is the frequency of a SNP site observed in a single cell as a proportion of all variant sorted cells.

Supplementary Figure 5

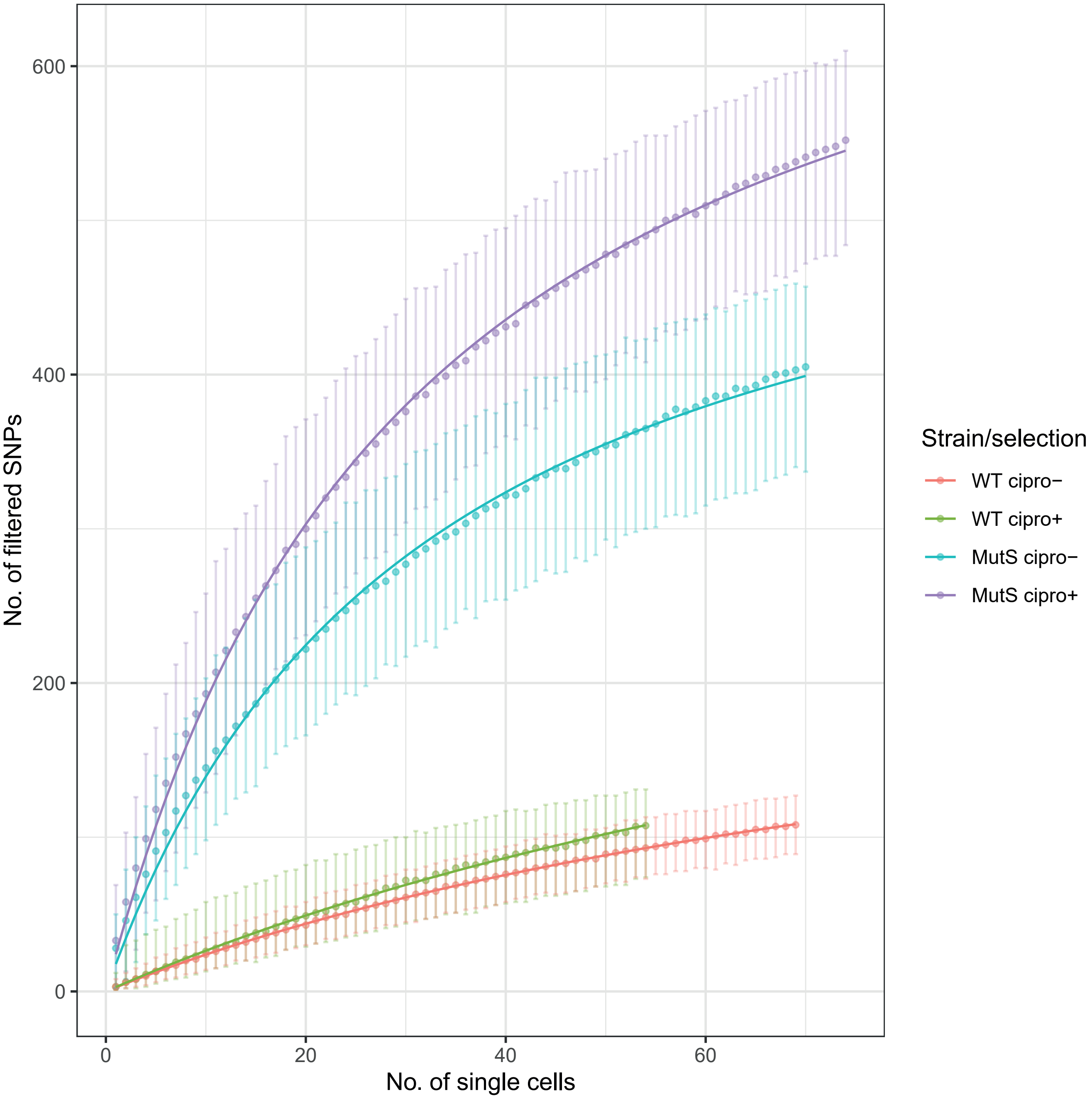


**Supplementary Figure 5** Rarefaction curves of identified SNPs in hypermutator and WT strains of *S.* Enteritidis grown in presence and absence of sub-inhibitory concentrations of ciprofloxacin. Total SNP diversity is estimated from the Michaelis-Menten curve prediction at infinite sample size although the curve failed to fit for the WT strain in the presence of ciprofloxacin. From these curve fits we obtained estimates of total SNP number as 8099.5 for WT without cipro. and 994.7 and 1202.48 for the hypermutator with and without cipro. respectively. The observed SNP numbers were 171, and 165 for the WT with and without cipro, and 539 and 714 for the hypermutator strains. Thus, we estimate that we obtained 2% of the total SNPs in the WT cipro -, and 54% and 59% of the SNPs for the hypermutator strains.

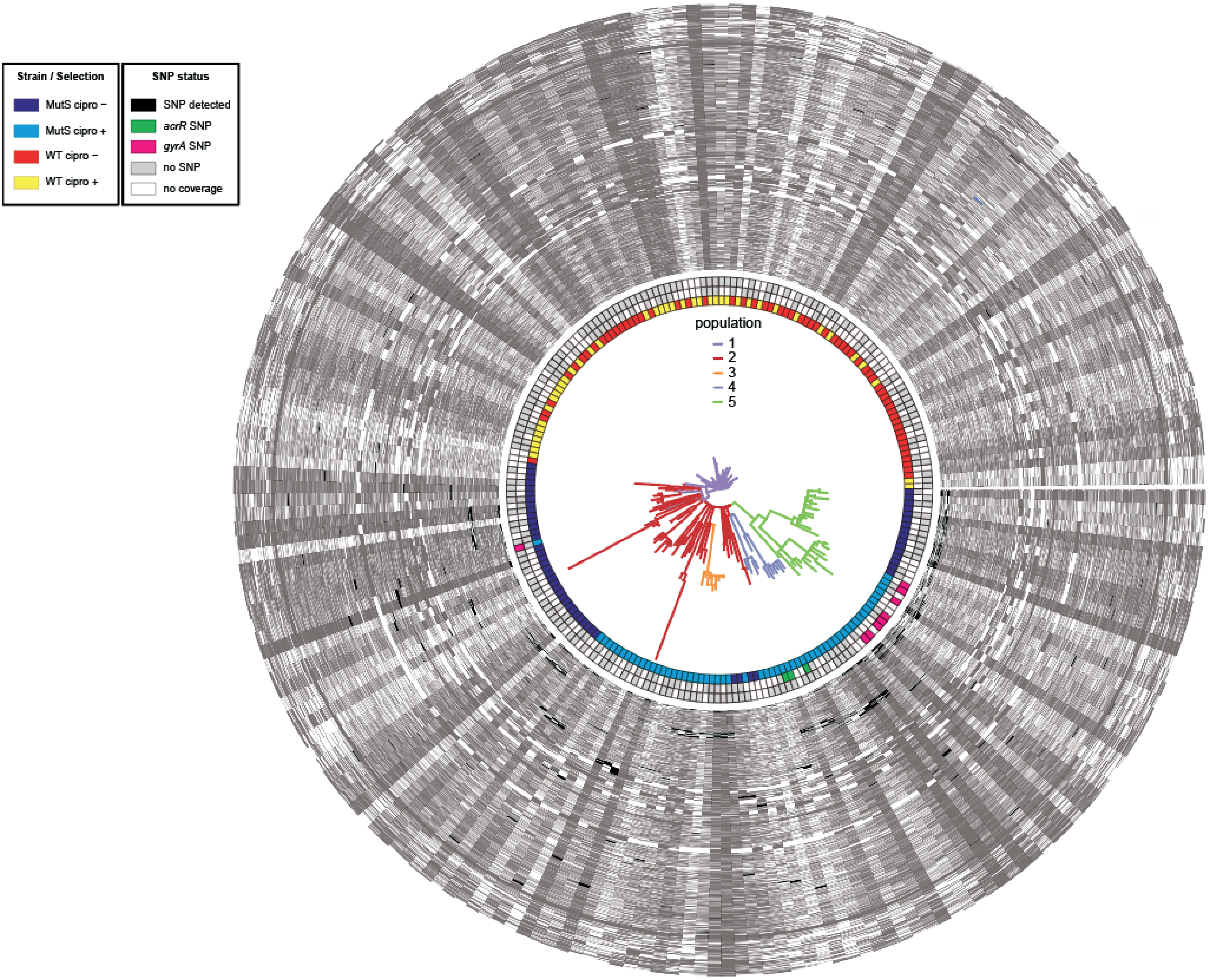


Supplementary Figure 6: Phylogeny and population structure of all single cell genomes evolved in the absence and presence of ciprofloxacin based on 1807 variant sites in coding and non-coding sites. The colour of the tree branche indicate the determined population group. Three rings outside the phylogeny highlight population group, and the presence of SNPs in acrR and gyrA respectively. The heatmap outside the phylogeny and inner rings indicates the coverage and SNP status of each site in each sample used to construct the phylogeny (black: SNP; grey: coverage but no variant and white: no coverage).

Supplementary Figure 7

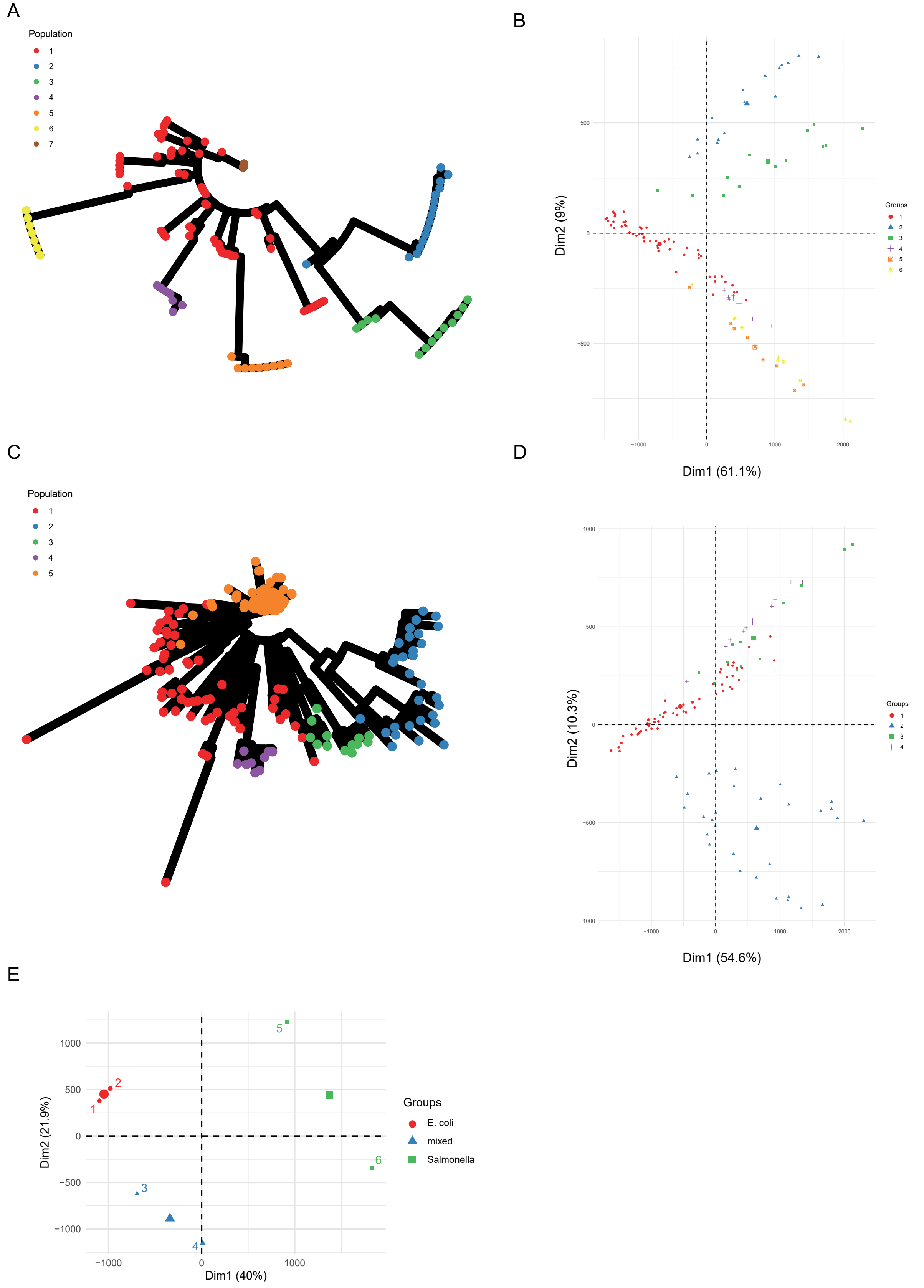


**Supplementary Figure 7** A) Phylogenetic tree and (B) PCA plot using 315 high confidence variant sites in coding regions. (C-D) as for A and B but using 1807 variant sites in coding and non-coding regions. PCA plots have basal populations (the least variant population identified in population analysis) removed to increase resolution. Populations are highlighted by the same colors in phylogenies and PCA plots. (E) PCA plot for mixed barnyard experiment (see Fig. 1B).
